# The lack of PPARα exacerbated the progression of non-alcoholic steatohepatitis in mice with spleen deficiency syndrome by triggering an inflammatory response

**DOI:** 10.3389/fimmu.2024.1381340

**Published:** 2024-04-03

**Authors:** Jiawen Huang, Jiayu Li, Yuan Peng, Tianqi Cui, Jingyi Guo, Siwei Duan, Kaili Zhou, Shangyi Huang, Jiabing Chen, Qincheng Yi, Min Qiu, Tingting Chen, Xiaoqin Wu, Chenlu Ma, Ziyi Zhang, Yi Zheng, Xi Tang, Yanqing Pang, Lei Zhang, Chong Zhong, Yong Gao

**Affiliations:** ^1^ Science and Technology Innovation Center, Guangzhou University of Chinese Medicine, Guangzhou, China; ^2^ Department of Pharmacy, Affiliated Hospital of North Sichuan Medical College, Nanchong, China; ^3^ Department of Phase I Clinical Research Center, The Second Affiliated Hospital of Guangzhou University of Chinese Medicine (Guangdong Provincial Hospital of Chinese Medicine), Guangzhou, China; ^4^ Department of Physiology and Pathophysiology, Tianjin Medical University, Tianjin, China; ^5^ Department of Hepatobiliary Surgery, The First Affiliated Hospital of Guangzhou University of Chinese Medicine, Guangzhou, China

**Keywords:** nuclear receptor, lipid metabolism, non-alcoholic steatohepatitis, inflammatory response, PPARα

## Abstract

**Background:**

In addition to abnormal liver inflammation, the main symptoms of non-alcoholic steatohepatitis (NASH) are often accompanied by gastrointestinal digestive dysfunction, consistent with the concept of spleen deficiency (SD) in traditional Chinese medicine. As an important metabolic sensor, whether peroxisome proliferator-activated receptor alpha (PPARα) participates in regulating the occurrence and development of NASH with SD (NASH-SD) remains to be explored.

**Methods:**

Clinical liver samples were collected for RNA-seq analysis. C57BL/6J mice induced by folium sennae (SE) were used as an SD model. qPCR analysis was conducted to evaluate the inflammation and metabolic levels of mice. PPARα knockout mice (PPARα^ko^) were subjected to SE and methionine–choline-deficient (MCD) diet to establish the NASH-SD model. The phenotype of NASH and the inflammatory indicators were measured using histopathologic analysis and qPCR as well.

**Results:**

The abnormal expression of PPARα signaling, coupled with metabolism and inflammation, was found in the results of RNA-seq analysis from clinical samples. SD mice showed a more severe inflammatory response in the liver evidenced by the increases in macrophage biomarkers, inflammatory factors, and fibrotic indicators in the liver. qPCR results also showed differences in PPARα between SD mice and control mice. In PPARα^ko^ mice, further evidence was found that the lack of PPARα exacerbated the inflammatory response phenotype as well as the lipid metabolism disorder in NASH-SD mice.

**Conclusion:**

The abnormal NR signaling accelerated the vicious cycle between lipotoxicity and inflammatory response in NAFLD with SD. Our results provide new evidence for nuclear receptors as potential therapeutic targets for NAFLD with spleen deficiency.

## 1 Introduction

Non-alcoholic fatty liver disease (NAFLD) is the most common chronic liver disease worldwide, affecting 12.5% of the global population (1). Non-alcoholic steatohepatitis (NASH) is the advanced stage of NAFLD, which typically increases the risk of developing into cirrhosis and hepatocellular carcinoma (HCC), ultimately leading to HCC (2). So far, NASH has become the fastest-developing cause of HCC. In clinical practice, changing lifestyle and drug therapy are the commonly used coping strategies mainly because there is currently no specific treatment method for NASH and the specific cause of NASH is not yet clear (3).

NASH is a chronic liver disease closely related to metabolic disorders, characterized by hepatocyte steatosis, inflammation, and fibrosis (4). Among them, inflammation is an important feature of NASH, which is a complex process caused by multiple factors. In brief, immune cells such as macrophages and T lymphocytes are activated by fatty acids and oxidative stress, releasing pro-inflammatory cytokines, leading to the progression of inflammation and fibrosis (5, 6). Secondly, abnormal fatty acid metabolism is one of the key factors in NASH inflammation. Once liver fatty acid metabolism is imbalanced, fatty acids accumulate excessively in hepatocytes, leading to the formation and enlargement of lipid droplets, thereby triggering an inflammatory response in the liver (7). Moreover, when exposed to excessive fatty acids and free radicals, hepatocytes can also result in increased oxidative stress, further increasing the inflammatory response and exacerbating the progress of NASH (8). In traditional Chinese medicine (TCM), spleen deficiency (SD) is the basis of NASH, which can be manifested as chest and rib swelling and pain, sighing, depression or irritability, lack of appetite, bloating, and loose stools (9). Mice gavaged with folium sennae were usually used as the SD model, exhibiting diarrhea, imbalance of short-chain fatty acid metabolism, and severe inflammation (10). However, it is not clear whether this severe inflammation will cause an inflammatory response in the liver and ultimately lead to metabolic disorders in the liver.

Peroxisome proliferator-activated receptors (PPARs) are ligand-activated transcription factors of the nuclear hormone receptor superfamily comprised of three subtypes, namely, PPARα, PPARβ/δ, and PPARγ, and they play a crucial role in glucose and lipid metabolism and inflammation (11). Among them, PPARα can promote the uptake and oxidation of fatty acids, inhibit adipocyte differentiation, and also promote the reverse transport of cholesterol by regulating the expression of related genes (12, 13). Although abnormal PPARα signaling is often associated with abnormal liver lipid metabolism, its abnormal expression is also accompanied by inflammatory reactions in the liver (14). Recent studies have begun to consider the crosstalk between hepatocytes and immunocytes. It is known that lipotoxicity caused by lipid metabolism disorders in hepatocytes can induce inflammatory reactions as shown in our previous study (15). The administration of the PPARα/γ dual agonist tesaglitazar had anti-inflammatory effects in ob/ob mice by reducing the number of pro-inflammatory adipose tissue macrophages (16). The current study investigated the hypothesis that SD-induced liver inflammation leads to abnormalities in the PPARα pathway, thereby accelerating susceptibility to NASH.

It is believed that once the inflammatory response intensifies, it will further induce lipid metabolism disorders to form a vicious cycle, ultimately exacerbating the development of the disease (17). Similar to this point of view, our previous study proved that hepatic Zbtb18 protein transcriptionally activates FAO and CLTC expression, which inhibits NLRP3 inflammasome’s activity, alleviating inflammatory stress and insulin resistance. In this study, WT mice and PPARα knockout (PPARα^ko^) mice gavaged with folium sennae (SE) were used as the SD model, a methionine–choline-deficient (MCD) diet was used as an incentive for NASH, and the molecular biology techniques and transcriptomics were utilized for detecting changes in inflammation, lipid metabolism, and PPARα expression.

## 2 Materials and methods

### 2.1 Animals

Six- to 8-week-old C57BL/6J mice were housed in an SPF-level breeding environment (24°C ± 2°C, a 12/12-h light/dark cycle) and had free access to water and food supervised by the Ethics Committee of Guangzhou University of Chinese Medicine. Mice were divided into the CON group and the SD group. SD mice were gavaged with 0.4 ml of 10% SE (w/v), while mice in the CON group were given the same equivalent normal saline. Seven days later, mice were executed, and the liver of each mouse was collected for further examination.

Six- to 8-week-old WT mice and PPARα knockout (PPARα^ko^) mice were fed with chow diet (CD) or MCD diet with SE gavage for 7 days. The serum and the liver of each mouse were collected for further detection.

### 2.2 Sample analysis

For qPCR, the total mRNA of each liver sample was extracted, followed by reverse transcription into cDNA, and then quantitative PCR was performed using Master Mix (ABclonal Co., Wuhan, China). The specific primers are shown in **Supplementary Table S1**.

For histopathologic analysis, liver samples were sliced after embedding and fixation. For immunohistochemistry, hematoxylin and eosin (H&E) staining kit, Oil Red O staining kit, and Sirius Red staining kit were used following the instructions. For immunohistochemistry (IHC), after dewaxing, hydration, and antigen repair, slices were blocked and incubated with antibodies overnight at 4°C, followed by incubation with the secondary antibody for 1 h, and pictures were observed using a microscope after being sealed.

### 2.3 Clinical sample and RNA-seq analysis

The liver samples of NAFLD and NAFLD-SD patients were collected from the First Affiliated Hospital of Guangzhou University of Chinese Medicine in accordance with the guidelines of the Declaration of Helsinki, and written informed consent was obtained from each patient supervised by the First Affiliated Hospital of Guangzhou University of Chinese Medicine Institutional Review Board and Ethics Committee.

For RNA-seq analysis, total RNA was extracted and RNA purity, quantification, and integrity were evaluated. Sequencing was conducted and the libraries were constructed according to the manufacturer’s instructions. The RNA-seq analysis was conducted by Berry Genomics Corporation (Beijing, China).

### 2.4 Statistical analysis

Data were shown as means ± SEM, and the significant differences were evaluated using Student’s *t*-test. A *p*-value <0.05 was considered statistically significant.

## 3 Results

### 3.1 PPAR signaling was abnormal in the liver of NAFLD-SD patients

**Figure 1:**
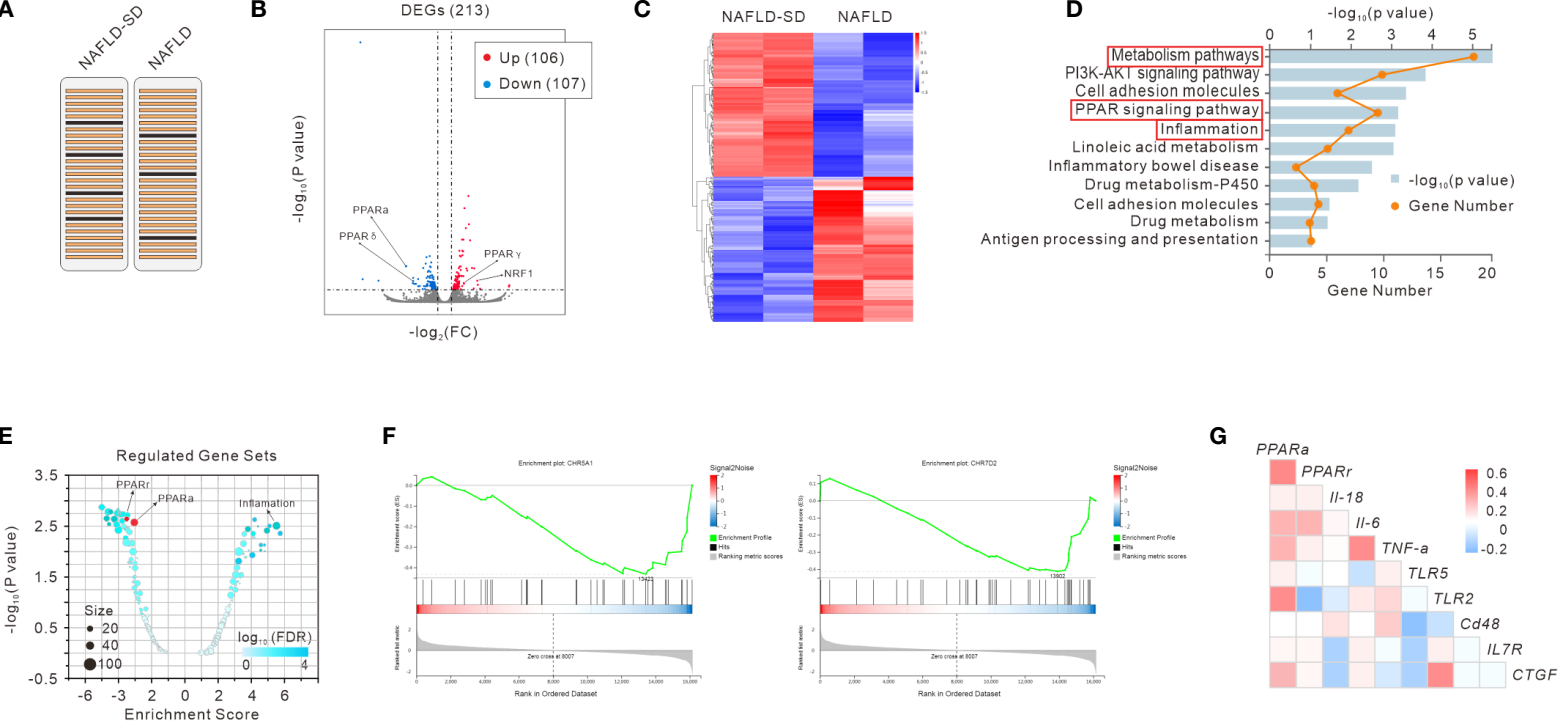
RNA-seq analysis of liver samples of NAFLD and NAFLD-SD patients **(A)**. Scatter plot **(B)**, heatmap **(C)**, and KEGG analysis **(D)** of RNA-seq data. Regulated gene sets **(E)**, gene set enrichment analysis (GSEA) **(F)**, and gene correlation analysis **(G)** of RNA-seq data.

To gain insight into the underlying molecular mechanisms of NAFLD and NAFLD-SD, we performed RNA-seq analysis of clinical liver samples from patients with NAFLD and NAFLD-SD (**Figure 1A**). The results showed that 213 genes were differently expressed with 106 upregulated and 107 downregulated differentially expressed genes (DEGs) (**Figure 1B**). RNA-seq data showed a significant difference between NAFLD-SD and NAFLD (**Figure 1C**), and the KEGG pathway analysis showed that the main differential pathways included metabolism and inflammation, with the PPAR signaling pathway closely associated with them (**Figure 1D**). In line with the KEGG analysis, gene set enrichment analysis (GSEA) showed that the DEGs (NAFLD-SD vs. NAFLD) were negatively correlated with the “PPAR signaling pathway” categories and were positively correlated with inflammation, suggesting that these changes could regulate the nuclear receptor and inflammation (**Figures 1E, F**). Pearson’s correlation analysis of the RNA-seq analysis data was performed to further explore the correlation between PPAR and genes involved in inflammation from the NAFLD-SD vs. NAFLD. The results showed that *PPARα* and *PPARr* mRNA levels were negatively correlated with the expression of the gene related to inflammation (**Figure 1G**).

### 3.2 The inflammatory response and fibrosis were increased in the liver of SD mice

**Figure 2:**
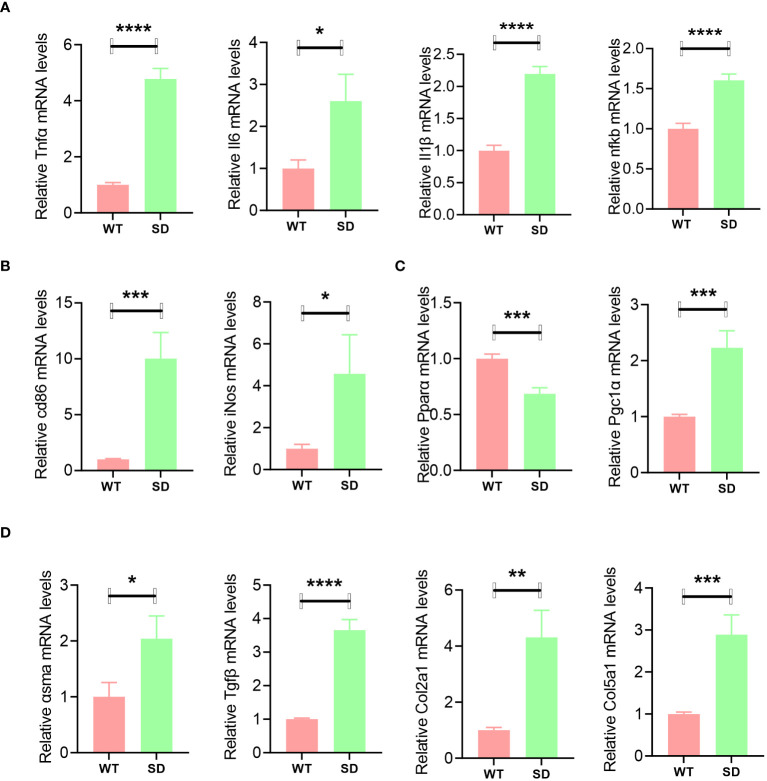
qPCR analysis of liver samples of WT and SD mice, including inflammatory genes such as *Il6*, *Tnfα*, *Il1β*, and *nfkb* **(A)**; macrophage marker genes *cd86* and *iNos* **(B)**; PPARα signaling-related genes such as *PPARα* and *Pgc1α* **(C)**; and fibrosis-related genes such as *asma*, *Tgfβ*, *Col2a1*, and *Col5a1* **(D)**. Data are shown as the mean ± SEM, *n* = 6. *p < 0.05, **p < 0.01, ***p < 0.005, and ****p < 0.001.

In order to explore the changes caused by SE-induced SD in the liver, qPCR analysis was conducted. The results showed that inflammatory genes such as *Il6*, *Tnfα*, *Il1β*, and *nfkb* were highly expressed in the SD group (**Figure 2A**). The macrophage infiltration marker genes such as *cd86* and *iNos* were also significantly increased in the liver of SD mice (**Figure 2B**). PPARα signaling-related genes were also detected, and it was found that *PPARα* was downregulated while *Pgc1α* was upregulated by SD intervention (**Figure 2C**). Moreover, the mRNA expression levels of *asma*, *Tgfβ*, *Col2a1*, and *Col5a1* were increased in the liver of SD mice, suggesting an increased tendency to liver fibrosis (**Figure 2D**).

### 3.3 The lack of PPARα exacerbated liver fibrosis and inflammatory response in NASH-SD mice

**Figure 3:**
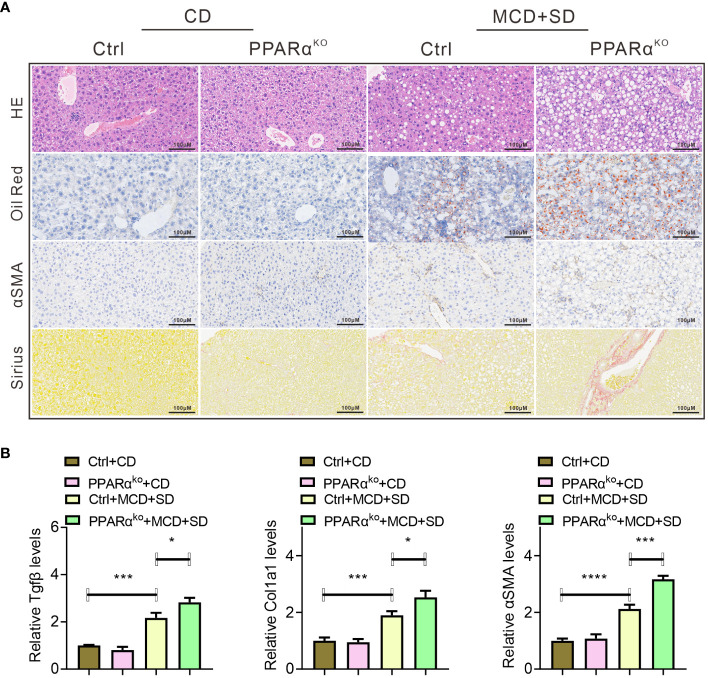
Histopathological analysis of liver samples of WT and PPARα^ko^ mice intervened with CD or MCD+SD, including H&E staining, Oil Red O staining, IHC of αSMA, and Sirius Red staining **(A)**. qPCR analysis of liver samples of those mice, including fibrosis-related genes such as *Tgfβ*, *Col1a1*, and *αSMA* **(B)**. Data are shown as the mean ± SEM, *n* = 6. *p < 0.05, ***p < 0.005, and ****p < 0.001.

PPARα^ko^ mice fed with MCD diet as well as gavaged with SE (MCD+SD) were used for further exploration. When fed with CD, there was barely any difference between WT and PPARα^ko^ mice in liver histopathological analysis, while when subjected to MCD+SD intervention, more severe pathological manifestations of lipid deposition and fibrosis were observed in the liver of PPARα^ko^ mice compared with WT mice (**Figure 3A**). Mice in the MCD+SD group exhibited high mRNA expression levels of *TGFβ1* and *Col1a1* in the qPCR results, with PPARα^ko^ mice exhibiting higher expression compared with WT mice (**Figure 3B**).

**Figure 4:**
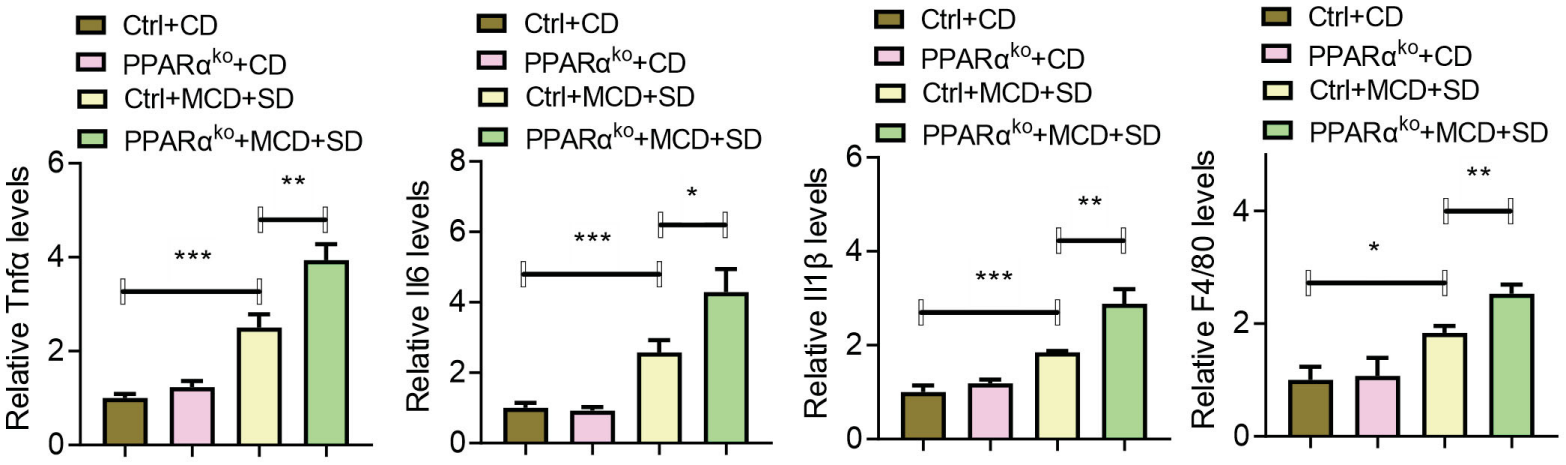
qPCR analysis of liver samples of WT and PPARα^ko^ mice intervened with CD or MCD+SD, including inflammatory genes such as *Il6*, *Tnfα*, *Il1β*, and *f4/80*. Data are shown as the mean ± SEM, *n* = 6. *p < 0.05, **p < 0.01, and ***p < 0.005.

Further experiments focused on the liver inflammation of those mice. When fed with CD, there was no significant difference in the expression levels of inflammatory genes including *Il6*, *Tnfα*, and *Il1β* as well as *F4/80* in the liver of both WT and PPARα^ko^ mice, while when intervened with MCD+SD, those genes in PPARα^ko^ mice were significantly higher than those in WT mice (**Figure 4**).

## 4 Discussion

The pandemic of NASH has forced us to consider countermeasures, and identifying susceptible populations of NASH and preventing their process in NASH are two of the feasible ways. TCM believes that SD, which is involved in the abnormal function of the spleen and liver as well as organs of the digestive system, is the key to the occurrence and development of NASH (18). An individual with SD is more likely to exhibit symptoms of digestive and metabolic abnormalities, which may trigger or accelerate NASH (19). Also, TCM research on NASH has always focused on SD (20). SE is a commonly used inducer for establishing SD models, and mice treated with its intervention may experience metabolic abnormalities, diarrhea, and other symptoms, which are typical manifestations of SD (21). This project outlines the increased susceptibility of SD individuals to NASH and points out one of its potential therapeutic targets—PPARα.

Inflammation is one of the important triggers in the progression of NAFLD to NASH (22). In this study, there are significant differences in metabolic and inflammatory pathways in the RNA-seq analysis of liver samples from NASH-SD and NASH patients, suggesting that metabolism and inflammation may be triggers for the development of SD toward NASH, as demonstrated by SD mice in our study. This is consistent with a recent study on increased inflammation and metabolic disorders in SD mice (10). However, SD-induced severe diarrhea in mice may also be associated with intestinal barrier dysfunction and dysbiosis of gut microbiota (23). For example, when transferred to sterile mice, specific microbial characteristics may be reshaped, and the progression of NASH can be delayed, indicating that mitochondrial dysfunction promotes the progression of NASH by exacerbating the gut–liver status as well as inflammation (24). Overall, the changes in the microenvironment of liver inflammation caused by different reasons will drive the activation of hepatic stellate cells and thereby accelerate the development of NASH, which is consistent with our research (25).

In NASH patients, not only severe inflammation and lipid deposition but also a tendency for liver fibrosis was observed (26). Similar results were observed in SD mice, indicating that individuals with SD are more susceptible to NASH. Meanwhile, NASH treatment strategies either seek to alleviate metabolic disorders and cell damage or directly target the accompanying inflammation and fibrosis, with targets for reducing the activation of inflammatory cascade reactions including nuclear receptor agonists (27). In this study, we observed that the liver of SD mice exhibited not only elevated levels of inflammation and a tendency toward fibrosis but also abnormal PPARα signaling, in which PPARα is one of the nuclear receptors. When entering the nucleus, PPARα can bind to promoters that are associated with β-oxidation-related factors such as ACADM and CPT1A and also enhance their transcriptional activity and ultimately drive the process of fatty acid β-oxidation (28). Also, activated PGC-1α can regulate fatty acid β-oxidation through synergism with transcription factors such as PPARα, indicating the crucial role that PPARα/PGC1α signaling plays in liver fatty acid metabolism homeostasis (29). PPARα is downregulated in many liver diseases such as NAFLD and NASH (30). In this study, we found for the first time that PPARα is downregulated in the liver of SD mice. Thus, the abnormal expression levels of PPARα and PGC1α occurred simultaneously with fibrosis and elevated inflammatory response in SD mice, indicating that this signaling may be the reason for the increased susceptibility of SD mice to NASH.

In further research, we investigated whether PPARα deficiency increased the susceptibility to NASH, using PPARα^ko^ mice and WT mice with MCD+SD intervention as the study subjects and conducting histopathological observations. H&E staining and Oil Red staining are commonly used to observe fat deposition in mouse liver, while Sirius staining and immunohistochemistry for detecting αSMA expression are commonly used to observe the severity of liver fibrosis (31). In our study, PPARα^ko^ mice treated with MCD+SD intervention showed more severe fat deposition and fibrosis in the above liver pathological analysis compared with WT mice, but there was no difference between the two in the CD diet. It is known that PPARα is highly expressed in the liver and participates in regulating fatty acid metabolism (32). After binding to fatty acid ligands, PPARα stimulates the transcription of genes containing PPARα response elements in its enhancer, with the most significant being those genes involved in lipid metabolism and energy homeostasis (33). Previously, Li’s research showed that there was no significant difference in the liver between normally fed PPAR^ko^ mice and WT mice, which is consistent with our research results (34). The absence of PPARα exacerbates the NASH performance caused by MCD+SD intervention, which is related to the functions of PPARα in regulating lipid metabolism homeostasis and inflammatory response. Meanwhile, our results also indirectly reflect that the absence of PPARα increases the susceptibility to NASH. In addition, only a preliminary exploration of the mechanism has been conducted, and the number of clinical samples used was limited, which cannot be representative of all situations. Further studies are needed in the future.

Overall, the lack of PPARα accelerated the vicious cycle between lipid metabolism and inflammatory response in NAFLD with SD. Similar results have been found in sepsis in which the metabolic and inflammatory responses to bacterial infection were impaired in the absence of PPARα, which leads to an enhancement in mortality due to bacterial sepsis (35). Our previous studies also exhibited that the ingredients of spleen-tonifying drugs can improve NAFLD or NASH by regulating PPAR signaling, which indirectly confirms the conclusion of this topic (36, 37). Unfortunately, there was no separate comparison between the MCD group and the MCD+SD group when setting up the groups, which will also be our next research direction. In addition, only a preliminary exploration of the mechanism has been conducted, and the number of clinical samples used was limited, which cannot be representative of all situations. Further studies are needed in the future.

## Data availability statement

The datasets presented in this study can be found in online repositories. The names of the repository/repositories and accession number(s) can be found below: https://www.ncbi.nlm.nih.gov/, PRJNA1061326.

## Ethics statement

The studies involving humans were approved by the First Affiliated Hospital of Guangzhou University of Chinese Medicine Institutional Review Board and Ethics Committee affiliated to Guangzhou University of Chinese Medicine. The studies were conducted in accordance with the local legislation and institutional requirements. The participants provided their written informed consent to participate in this study. The animal study was approved by the Ethics Committee of Guangzhou University of Chinese Medicine affiliated to Guangzhou University of Chinese Medicine. The study was conducted in accordance with the local legislation and institutional requirements. Written informed consent was obtained from the individual(s) for the publication of any potentially identifiable images or data included in this article.

## Author contributions

JH: Writing – review & editing, Writing – original draft, Investigation, Funding acquisition, Formal analysis. JL: Writing – review & editing, Writing – original draft, Investigation, Formal analysis. YP: Writing – original draft, Writing – review & editing, Formal analysis. TQC: Writing – original draft, Visualization, Investigation. JG: Writing – original draft, Visualization, Investigation. SD: Writing – original draft, Software, Investigation. KZ: Writing – original draft, Software, Investigation. SH: Writing – original draft, Investigation, Data curation. JC: Writing – original draft, Investigation, Data curation. QY: Writing – original draft, Investigation. MQ: Writing – original draft, Investigation. TTC: Writing – original draft, Investigation. XW: Writing – original draft, Investigation. CM: Writing – original draft, Investigation. ZZ: Writing – original draft, Investigation. YZ: Writing – original draft, Investigation. XT: Writing – original draft, Investigation. YQP: Writing – review & editing, Conceptualization, Funding acquisition. LZ: Writing – review & editing, Writing – original draft, Resources, Formal analysis. CZ: Writing – review & editing, Writing – original draft, Resources, Formal analysis. YG: Writing – review & editing, Writing – original draft, Resources, Funding acquisition, Formal analysis, Conceptualization.
