# Supplementary material for: The lack of PPARα exacerbated the progression of non-alcoholic steatohepatitis in mice with spleen deficiency syndrome by triggering an inflammatory response
Source: Front Immunol. 2024 Apr 3;15:1381340. doi: 10.3389/fimmu.2024.1381340 (PMC11021588; doi:10.3389/fimmu.2024.1381340)
Supplement: Supplementary file 1 [file DataSheet_1.docx]

| **eGene** | **Forward Primer** | **Reward Primer** |
| --- | --- | --- |
| *Tnfα* | CCCTCACACTCAGATCATCTTCT | GCTACGACGTGGGCTACAG |
| *II6* | TAGTCCTTCCTACCCCAATTTCC | TTGGTCCTTAGCCACTCCTTC |
| *II1β* | GCAACTGTTCCTGAACTCAACT | ATCTTTTGGGGTCCGTCAACT |
| *nfkb* | ATGGCAGACGATGATCCCTAC | TGTTGACAGTGGTATTTCTGGTG |
| *cd86* | TGTTTCCGTGGAGACGCAAG | TTGAGCCTTTGTAAATGGGCA |
| *INOS* | GTTCTCAGCCCAACAATACAAGA | GTGGACGGGTCGATGTCAC |
| *Pparα* | AGAGCCCCATCTGTCCTCTC | ACTGGTAGTCTGCAAAACCAAA |
| *Pgc1α* | TATGGAGTGACATAGAGTGTGCT | CCACTTCAATCCACCCAGAAAG |
| *αsma* | GTCCCAGACATCAGGGAGTAA | TCGGATACTTCAGCGTCAGGA |
| *Tgfβ* | CTCCCGTGGCTTCTAGTGC | GCCTTAGTTTGGACAGGATCTG |
| *Col2a1* | GGGAATGTCCTCTGCGATGAC | GAAGGGGATCTCGGGGTTG |
| *Col5a1* | CTTCGCCGCTACTCCTGTTC | CCCTGAGGGCAAATTGTGAAAA |
| *F4/80* | CCCCAGTGTCCTTACAGAGTG | GTGCCCAGAGTGGATGTCT |
| *β-actin* | GGCTGTATTCCCCTCCATCG | CCAGTTGGTAACAATGCCATGT |

**Table S1. Antibody information for western bolt analysis**

**
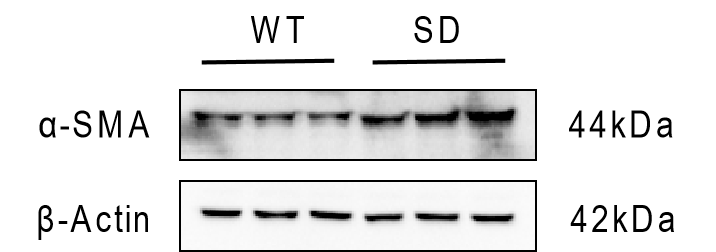
**

**Supplementary Figure 1**

The expression of α-SMA protein level detected by western blot.

**
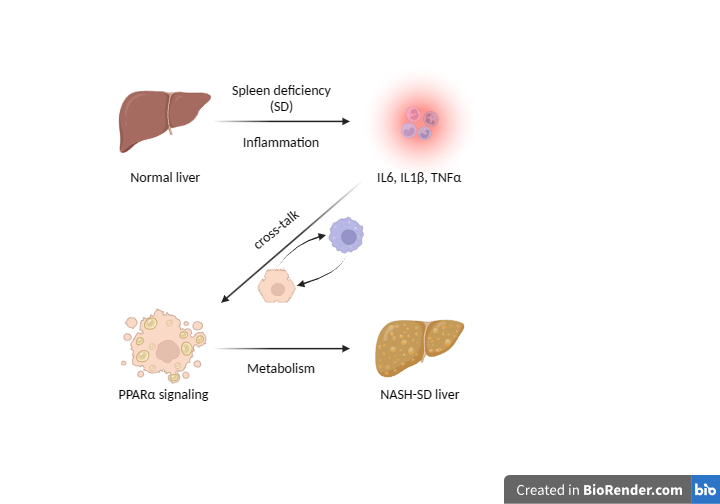
**

**Supplementary Figure 2**

Graphical abstract (Created with BioRender.com).
